# Anesthetic management for a patient with aortic stenosis who underwent transcatheter aortic valve implantation after introduction of percutaneous cardiopulmonary support

**DOI:** 10.1186/s40981-018-0168-z

**Published:** 2018-04-04

**Authors:** Takashi Kobayashi, Shohei Ogawa, Kenji Suzuki

**Affiliations:** Department of Anesthesiology, School of Medicine, Iwate Medical University, 19-1 Uchimaru, Morioka-shi, Iwate, 020-8505 Japan

**Keywords:** Transcatheter aortic valve implantation, Low output syndrome, Percutaneous cardiopulmonary support

## To the Editor

### Correspondence/findings

The patient was a 74-year-old male with a height of 155 cm and a body weight of 41 kg. At the age of 63, he underwent off-pump coronary artery bypass grafting due to a previous myocardial infarction. The patient had been hospitalized several times due to heart failure secondary to aortic stenosis (AS) since approximately 71 years of age. On transthoracic echocardiography, the patient’s left ventricle showed diffuse hypokinesis and an ejection fraction of 27%. The aortic valve was highly calcified and showed mobility restrictions together with the tricuspid valve; moderate to severe AS was also present.

**Fig. 1:**
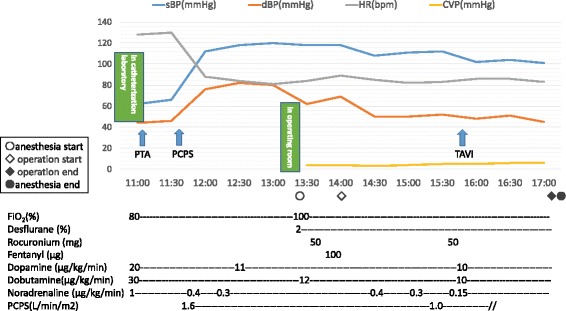
Perioperative clinical course

While planning the surgery, the heart team at our institution considered the patient’s cardiovascular dysfunction and history of open-chest surgery and decided that conventional aortic valve replacement would be a high-risk procedure. Therefore, we planned to perform transcatheter aortic valve implantation (TAVI). However, the patient had to wait for insurance approval for a self-expandable valve (CoreValve®). He underwent balloon aortic valvuloplasty (BAV) three times while awaiting insurance approval for the CoreValve®. The last BAV was performed 3 months before hospitalization, which controlled his heart failure until insurance approval. His condition seemed to be stable, but in the process, he developed orthostatic breathing and low output syndrome (LOS) and was transferred from the general ward to the intensive care unit (ICU). Tracheal intubation was performed by an ICU physician. The patient was administered 3 mg of midazolam and 40 mg rocuronium intravenously. We believed his condition to be a result of overhydration due to his decreased urine output after hospitalization. Despite administering intravenous dopamine, dobutamine, and noradrenaline at high doses, the patient’s vital signs were unstable. Therefore, it became necessary to perform an emergency TAVI after introducing percutaneous cardiopulmonary support (PCPS). The patient was inserted a PCPS catheter from the right femoral artery and right femoral vein and then started thigh-femoral PCPS. Because the patient’s vital signs were stable, he was then transferred to the operating room. General anesthesia was maintained by inhalation of 2% desflurane, with sufficient administration of rocuronium. Just before surgery, 100 μg of fentanyl was administered. A self-expanding 29-mm TAVI valve CoreValve® was placed on top of the aortic valve. Although the patient’s arterial pressure decreased during the CoreValve® expansion, it quickly recovered after full expansion. The perioperative course is shown in Fig. 1.

On transthoracic echocardiography, the patient’s left ventricular function did not change compared with the preoperative condition, but the mobility of the implanted artificial valve was good.

Since TAVI became covered by health insurance, it has gained wide clinical use in our country. It is anticipated that TAVI will become more popular in the future [1].

However, patients scheduled for TAVI are in critical condition. PCPS is effective in the treatment of circulatory failure secondary to valvular heart disease or heart failure [2]. In this case, it is important to determine whether the patient can withstand the reduction in blood pressure accompanying general anesthesia introduction and valve expansion due to low left ventricular function. Regarding the low left ventricular function, the patient’s intraoperative vital signs were stabilized by circulatory assistance by PCPS. During anesthesia, we tried to maintain a sufficient preload to preserve the PCPS flow. To maintain the patient’s blood pressure, we used a high dose of inotropic agents. There have been reports using supplementary circulation such as PCPS at the time of a sudden change in preventive or intraoperative disorder [3–6], but we could not identify any report describing the introduction of PCPS after a sudden change in the condition of a patient with AS and the urgent implementation of TAVI. We believe that the emergent TAVI following PCPS is rare and that most Japanese anesthesiologists are not familiar with this type of perioperative management. In Japan, TAVI is difficult to perform at the emergent setting, as the decision for the indication and preparation of several devices cannot be made in a short time. In this case, this patient was scheduled for TAVI; therefore, it was fortunate that the device was ready.

PCPS is useful for the circulatory management of LOS, making it possible to treat the original disease after saving the patient’s life.

## Abbreviations

AS: Aortic stenosis
BAV: Balloon aortic valvuloplasty
ICU: Intensive care unit
LOS: Low output syndrome
PCPS: Percutaneous cardiopulmonary support
TAVI: Transcatheter aortic valve implantation
